# Construction of Infectious Clones of Begomoviruses: Strategies, Techniques and Applications

**DOI:** 10.3390/biology10070604

**Published:** 2021-06-29

**Authors:** Mohd Faiz Mat Saad, Aziz Ramlee Sau, Muhamad Afiq Akbar, Syarul Nataqain Baharum, Ahmad Bazli Ramzi, Noraini Talip, Hamidun Bunawan

**Affiliations:** 1 Institute of Systems Biology, Universiti Kebangsaan Malaysia, Bangi 43600, Selangor, Malaysia; faizsaad@ukm.edu.my (M.F.M.S.); azizramleesau@gmail.com (A.R.S.); nataqain@ukm.edu.my (S.N.B.); bazliramzi@ukm.edu.my (A.B.R.); 2 Department of Biological Sciences and Biotechnology, Faculty of Science and Technology, Universiti Kebangsaan Malaysia, Bangi 43600, Selangor, Malaysia; muhdafiq.akbar@gmail.com (M.A.A.); ntalip@ukm.edu.my (N.T.)

**Keywords:** begomovirus, infectious clone, strategy, geminiviridae

## Abstract

**Simple Summary:**

*Begomovirus* has a wide host range and threatens a significant amount of economic damage to many important crops such as tomatoes, beans, cassava, squash and cotton. There are many efforts directed at controlling this disease including the use of insecticides to control the insect vector as well as screening the resistant varieties. The use of synthetic virus or infectious clones approaches has allowed plant virologists to characterize and exploit the genome virus at the molecular and biological levels. By exploiting the DNA of the virus using the infectious clones strategy, the viral genome can be manipulated at specific regions to study functional genes for host–virus interactions. Thus, this review will provide an overview of the strategy to construct infectious clones of *Begomovirus*. The significance of established infectious clones in *Begomovirus* study will also be discussed.

**Abstract:**

Begomovirus has become a potential threat to the agriculture sector. It causes significant losses to several economically important crops. Given this considerable loss, the development of tools to study viral genomes and function is needed. Infectious clones approaches and applications have allowed the direct exploitation of virus genomes. Infectious clones of DNA viruses are the critical instrument for functional characterization of the notable and newly discovered virus. Understanding of structure and composition of viruses has contributed to the evolution of molecular plant pathology. Therefore, this review provides extensive guidelines on the strategy to construct infectious clones of *Begomovirus*. Also, this technique’s impacts and benefits in controlling and understanding the *Begomovirus* infection will be discussed.

## 1. Introduction

The first infectious clones of bacteriophage QB was reported in 1978 and this was achieved by inserting the cDNA genome into a plasmid vector [1]. In 1980, the infectious clone of retroviral DNA was constructed [2] and was followed by cDNA of poliovirus genome that was transfected into the mammalian cell in the following year [3]. With these notable discoveries, members of infectious DNAs of almost all virus families have been reported. The construction of infectious clones of plant viruses has been established by plant virologists. To characterise a virus at the molecular level, constructing an infectious clone is necessary to study viral replication, range of host species, movement, pathogenicity, and genomic regions’ roles [4]. However, the construction of an infectious clone that involves the cloning of many subgenomic cDNA fragments is frequently laborious [5]. The construction of an infectious clone is still gruelling and many obstacles remain including unpredictability and toxicity in bacterial host–virus sequences [6,7].

The family Geminiviridae consists of nine genera that mainly affect a wide range of dicotyledonous plants. Of all the nine genera, *Begomovirus* is the largest member of the group, which has about 322 species. Begomoviruses have either monopartite or bipartite genome. It can only be infecting dicots plants through whitefly (*Bemisia tabaci*) as the vector. This virus is present in both Old World (both genomes) and the New World (mainly bipartite genome, with one describes as monopartite genomes recently) [8,9]. Bipartite begomoviruses encode seven or eight proteins, while monopartite viruses encode only five or six proteins. Alphasatellites, betasatellites and deltasatellites are three types of circular DNA satellites related to the begomoviruses [10,11]. The International Committee on Taxonomy of Viruses (ICTV) recently revised the International Code of Virus Classification and Nomenclature to allow satellite nucleic acid classification. This alteration creates *Tolecusatellitidae*, a brood of single-stranded DNA satellites linked with begomoviruses [12].

**Figure 1:**
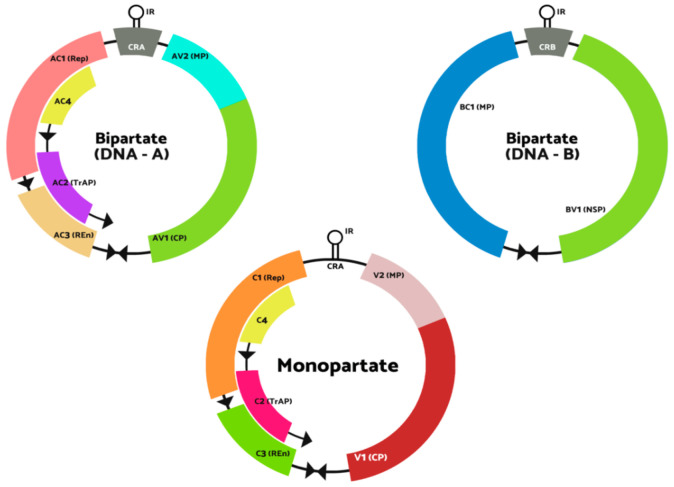
Genomic structure of begomoviruses. The ORFs are labelled as V if it encodes the virion-sense genes and labelled C if it encodes in complementary-sense genes. According to the genome components, the bipartite genome (top) is tagged with A or B, DNA-A or DNA-B. The two shared “common regions” CRA and CRB genomic components of bipartite viruses are shown in grey inside the intergenic region (IR). The stem-loop position in the IR that contains the conserved TAATATTAC sequence is shown. CP, coat protein; MP, movement protein; NSP, nuclear shuttle protein; REn, replication enhancer protein; Rep, replication-associated protein; TrAP, transcriptional activator protein.

Bipartite *Begomovirus* contains two components in their genomes: the DNA-A and DNA-B with a size of 2.5–2.6 kb. DNA-A is the part that makes proteins responsible for functions like replicating the virus, encapsidation of both genomic molecules and viral gene expression regulation. However, DNA-B creates the protein responsible for the mechanism of viral DNA molecules in and among the host cells [13]. DNA-A components in the genomes can replicate autonomously. However, it needs DNA-B components for systemic infection to occur. The intergenic region of DNA-A and DNA-B consists of about 200 bp of conserved sequence (Common Region (CR)), including the TAATATTAC sequence v-*ori* in the conserved stem-loop. The monopartite *Begomovirus* genome organization resembles the DNA-A component in the bipartite *Begomovirus* genome [14]. Figure 1 shows the structure genomes of *Begomovirus* (adapted from the 10th online report of the International Committee on Taxonomy of Viruses (ICTV)).

There are two components in the genomes of bipartite *Begomovirus*: DNA-A and DNA-B, whereas monopartite *Begomovirus* only contains one genome that resembles DNA-A. DNA-A and monopartite *Begomovirus* genome are made up of 6 open reading frames encoded for AV1 and AV2 in the virion strand. In contrast, in the complementary strand, there are four encoded proteins, which are AC1, AC2, AC3 and AC4 [14]. In general, AC1 protein is called the replication-associated protein (Rep), which functions in initiating viral DNA replication through the binding of the protein to the iterated motifs (iterons) and introduces a nick to the conserved region (CR) in the intergenic region (IR) [15,16]. According to Arguello-Astroga et al. [17], the Rep protein also coheres to the plant homolog of retinoblastoma protein (Rb) to support the viral DNA replication host factor. Sunter and Bisaro [18] and Bisaro [19] have reported that AC2 protein, which is known as transactivactional activation protein (TraP) that transactivates the expression of viral senses gene expression in DNA-A and B. It also helps in the regulation of transcriptional and post-transcriptional gene silencing (PTGS). AC4 protein plays a vital role as a symptom determinant, which affects cell-cycle control. AC4 protein also counters the host response towards the Rep expression [20]. The DNA-B compartment has two encoded proteins, which are translated from one of the virus strands and the other is from the complementary strand. The BV1 protein is a nuclear-shuttle protein, while BC1 is a movement protein [21,22].

Although the etiological agents causing *Begomovirus* were whiteflies under natural conditions, it is necessary to create artificial infections with cloned *Begomovirus* DNAs to study the various aspects of the virus, including the function of gene and virus-plant interactions [23]. Therefore, this review describes methods that are widely used to prepare and test infectious clones for different begomoviruses. The strategy to construct the full-length infectious clones of *Begomovirus* will be summarised. Also, the importance of infectious clones in understanding the *Begomovirus* infections and functional genomics will be highlighted.

## 2. Full-Length Infectious Clones Construction Strategy

**Figure 2:**
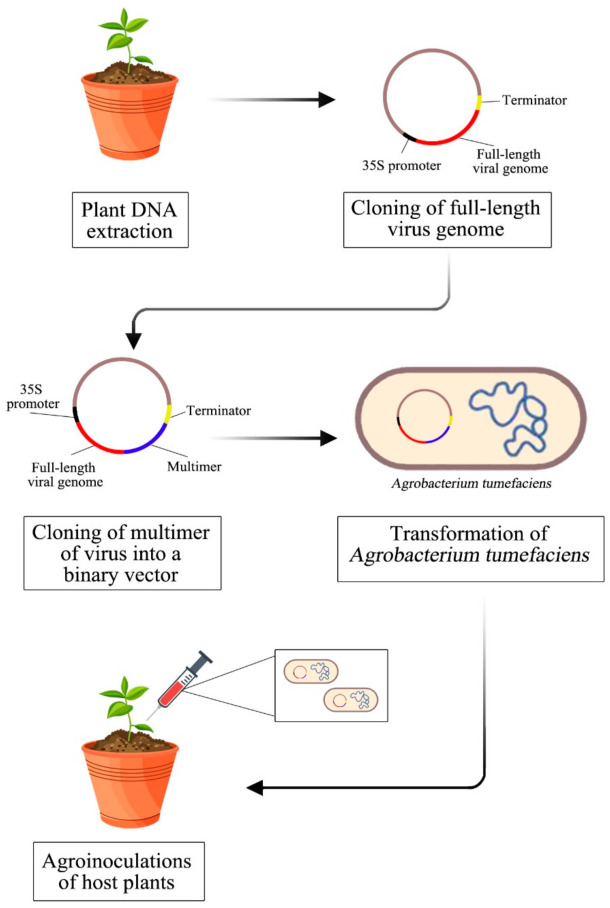
Summary of the methodology of constructing an infectious clone for *Begomovirus*.

An infectious clone is necessary for researchers to study viruses at the molecular and biological levels. An infectious clone has the capability to exploit the viral genome. Mutagenesis of specific regions can be introduced to study the coding part of the genome for studies on host–virus interactions. Figure 2 shows a summary of the methodology used to construct an infectious clone of begomoviruses. Starting from the total plant DNA isolation to obtain the viral genome, this is followed by the viral genome’s amplification. Next is cloning a full-length virus genome and multimer to produce a complete construct of an infectious clone. Finally, transformation into *Agrobacterium tumefaciens* and transfection to host plants for constructing an infectious clone for *Begomovirus*.

### 2.1. Isolation of Total Plant DNA

The first stage of constructing an infectious clone is the isolation of the genome of the virus. Through the extraction of host symptomatic plant total DNA, the viral genome can be obtained. Several protocols have been used widely to produce high-quality and sufficient amounts of DNA. The established protocol by Dellaporta et al. [24], Doyle and Doyle [25], and Palmer et al. [26] are examples of protocols that are well-known. In recent years, the advancement in technology has led to the development of rapid and straightforward kits to extract total plant DNA such as DNeasy Plant Mini Kit (Qiagen, Hilden, Germany), AuPrep^TM^ DNA extraction kit (Life Technologies, Carlsbad, CA, USA), Nucleospin^®^ Plant II total DNA purification kit (Macherey-Nagel, Düren, Germany) and Gentra Puregene DNA Reagents (QIAGEN, Valenia, CA, USA).

### 2.2. Amplification of Viral Genomes

The next step to produce a full-length infectious clone is the amplification of the complete viral genome from the total plant DNA extracts. There are two commonly used amplification practices for begomoviruses, which are the polymerase chain reaction (PCR) and rolling circle amplification (RCA) [27]. In a standard PCR method, an overlapping primer set needs to be constructed [28]. RCA is an isothermal enzymatic method to amplify a short DNA or RNA primer, forming a lengthy single-stranded DNA or RNA using a circular DNA template and unique *phi*29 polymerase (Templiphi™, GE, Healthcare). The amplification product would be a long single-stranded DNA with repeating bases that will complement the template. RCA is an advantageous technique to construct infectious clones for *Begomovirus* [29,30]. RCA is more advantageous than PCR as cloning sequence information is unnecessary, cost-effective and less laborious [31].

### 2.3. Cloning of Multimer and Full-Length Genome

In general, due to the size of the small genomes of *Begomovirus*, the cloning of the full genome is relatively easy. To produce a tandem repeat that comprises two origins of replication (V-*ori*), the construction of infectious clones of *Begomovirus* require more than one mer (unit) of the genome to boost their ability to infect the host cells [32]. From the amplification through the RCA methods that produce a circular ss-DNA (1.0 mer), a specific restriction enzyme (RE) will be used to linearize the circular DNA. The linearized product will be subsequently being inserted and ligated into a plasmid such as p-GEM-T Easy (Promega), pBlueScript (Stratagene), PTZ57R/T (Fermentas), Pjet1.2/blunt (ThermoFisher) and others that have been treated with the same RE. The plasmid + 1.0 mer were then treated with another RE to produce a multimer (0.01 mer–0.9 mer) that includes the origin of replication (V-*ori*). The multimer is then transformed into a destination vector and subsequently, a full length (1.0 mer) of the viral genome is ligated together in the destination vector to produce (destination vector + multimer + 1.0 mer).

### 2.4. Transformation into Agrobacterium Tumafaciens

There are three methods commonly used to transform genes of interest into *Agrobacterium tumefaciens*: electroporation, the freeze/thaw method and triparental mating [33]. The most efficient way of inserting a gene of interest into *Agrobacterium tumefaciens* is electroporation. The electroporation method uses electricity to create aqueous pores in the lipid membrane of bacteria. The pores formed are sufficient enough to allow the pass-through of DNA molecules to enter the cells. The regulation of electric field strength and duration of the pulse and the plasmid size determine this method’s efficiency [34,35]. Previously, research by Nair et al. [36] has reported successfully introduce a 200 kb plasmid into *Agrobacterium tumefaciens*.

As an alternative form of transforming *Agrobacterium tumefaciens* with foreign DNA, a freeze/thaw technique is considered a fast and straightforward method [37,38]. Based on this technique, the uptake of foreign DNA relies on the damage of the cell wall caused by the exposure to the rapid changes of temperature that alter the membrane fluidity of the bacterial cells [39]. This freeze/thaw technique is widely used as it does not require specialized equipment [33].

Triparental mating is an effective method for cloning a mobilized plasmid into *Agrobacterium tumefaciens*. This technique uses two *Escherichia coli* strains to transfer the plasmid carrying the gene of interest into *Agrobacterium tumefaciens*. The first strain of *E. coli* has a self-transmissible plasmid, also known as the helper plasmid, as it encodes for the protein that helps in the formation of the mating bridge and the transfer process of itself or another mobilized plasmid to the recipient cells. Once mating is secure, the helper plasmid will transfer itself into the second *E. coli* strain called the donor strain, which carries the plasmid of interest. Trans-acting functions of the helper plasmid act to transform the plasmid of interest by the donor strain into the *Agrobacterium tumefaciens* cells [40]. Table 1 below shows the strategy used by geminivirologists in producing infectious clones of different *Begomovirus*.

### 2.5. Agroinoculation of Host Plant

Agroinoculation is a technique that allows the transmission of *Begomovirus* infectious clones into various host plants with the aid of *Agrobacterium tumefaciens* [95,96]. This method is cost-effective and more straightforward to employ than biolistic inoculation, which required sophisticated equipment such as the particle gun [97]. According to this method, the culture of *Agrobacterium tumefaciens,* which carries the plasmid of interest, is plated on agar with antibiotic selection specific to the plasmid of interest. The positive growing colonies are selected and grown in a liquid culture containing the antibiotic for further selection. The growing bacteria are then harvested and resuspended in sterile distilled water before subject to the absorbance reading at 600 nm (OD_600_). A syringe is used to infiltrate the abaxial side of selected plant leaves. A needle is used to incise a small hole beforehand to create a wound. A needled syringe containing *Agrobacterium tumefaciens* culture bearing the plasmid of interest is used to inoculate the bacteria culture to the side of the plant’s incision. The infiltrated plant is incubated for a short period (1–4 days) before conducting the microscopy analysis. In general, it is best to use the immature plants for agroinoculation as they are more prone to infection with *Agrobacterium*. Alternative to agroinoculation, the biolistic method by which host plants are bombarded with the DNA containing-gold particles has been used in several studies of *Begomovirus* infectivity [43,72,89,91].

## 3. The Significance of Established Infectious Clones in the Begomovirus Study

The construction of an infectious clone allows functional analysis of the viral at the molecular level and their biological roles in replication, pathogenesis and transmission [32,98]. Creating infectious clones that contain wild-type viral genomes enables the establishment of an inoculum that can be used for resistance screening, this is necessary to avoid a genetically inconsistent viral population. Also, this eliminates the need for the maintenance and passage of the virus within plants. Infectious clones also provide an alternative to ineffective inoculation procedures such as grafting and infections transmitted via insect vector [99]. Infectious clones can be altered to achieve virus-induced gene silencing (VIGS) without the need for stable plant transgenesis as well as expression vectors for the plant where it can be applied in plant pathology and used in plant gene function study [100,101]. Despite this, the ability to reconstitute the virus that is fully functional and transmissible will lead to the uncontrolled use of infectious clones that may pose a risk to the environment. These risks could arise due to their inherent pathogenicity and the effect of any introduced genetic modifications [99].

In *Begomovirus*, the construction of infectious clones has enabled the acquisition of a huge amount of knowledge. Artificial manipulation of *Begomovirus* genomes such as site-directed mutagenesis, deletion or insertion and rearrangement have reported gaining new insights related to virus-host cell interactions. Infectious clones in the genus *Begomovirus* also essential for screening resistant varieties.

### 3.1. Functional Study on the Begomovirus Genomes Using Infectious Clones

Infectious clones for plant viruses are necessary tools in molecular virology as the cloned genome is easily manipulated. The characterization of a virus is possible by generating infectious clones [102].

#### 3.1.1. V2 Protein Functional Analysis

In monopartite *Begomovirus*, V2 plays its role as movement protein, whereas in bipartite *Begomovirus*, its function is being replaced by the BC1 protein of DNA-B. Infectivity analysis of *Tomato leaf curl Palampur virus* (ToLCPalV) and its AV2 mutants of ToLCPalV have revealed that AV2 protein is necessary for symptoms development such as leaves curling down, epinasty and chlorotic leaves. AV2 protein of *Okra enation leaf virus* (OELV) also reported to function as symptomatic induction, the OELV AV2 mutants cannot infect *Nicotiana benthamiana*, no necrosis or leaf-curling symptom observed [103,104].

#### 3.1.2. Functional Studies of C1 Protein

Replication-initiator protein (Rep) encoded by the AC1 gene is vital for virus replication. In the *African Cassava mosaic virus* (ACMV), Rep protein has been reported to contribute to viral infections [105]. Throughout Rolling Circle Replication (RCR), Rep will cleave the strand of virion-sense in a common region inside the mononucleotide sequence and secure it after one round of replication, similar actions as an endonuclease and nucleotidyltransferase [15,106]. A potential cyclin interaction motif (RXL) in the ACMV Rep sequence can be an alternative cycle controls to recognise interaction with retinoblastoma protein (pRBR) within plant homologs. The RXL motif also is necessary for *Nicotiana benthamiana* plants’ virus infection in producing the symptoms {105].

#### 3.1.3. Functional Studies of C2 Protein

Elucidation of the role of C2 protein in symptom determination and replication in the *Bhendi yellow mosaic virus* (BYMV) has been conducted through the construction of C2 mutants in BYMV. Two stop codons were inserted into the ORF that encodes the C2 protein. The host plant inoculated with BYMV bearing a mutated C2 protein displays the suppression of symptoms and a decrease in viral load. This suggests that BYMV C2 protein is vital in infection as it involves symptom determination and the viral replication process [107]. However, in different *Begomovirus*, C2 protein may play a different role. Stanley et al. [108] reported the C2 protein in *Beet curly top virus* (BCTV) does not play any role in infectivity. In contrast, a study conducted by Baliji et al. [109] in *Spinach curly top virus* (SCTV) has suggested that C2 mutant of SCTV produces a milder symptom of infection, which in turn indicates that C2 protein is required for infectivity.

#### 3.1.4. C4 Protein Functional Analysis

In general, ORF encodes for AC4 protein in *Begomovirus* function in the development of symptoms. The loss of C4 function leads to the attenuation of the symptom and reduced infectivity [110,111,112,113]. In bipartite *Begomovirus*, the function of AC4 protein is less conserved as compared to monopartite *Begomovirus*. This is shown by the alignment of the AC4 region sequence of bipartite *Begomovirus*, indicating that the sequence is less conserved. Bipartite *African cassava mosaic virus* with AC4 mutants (ACMV) expressed symptoms and infection [114,115]. While in *Tomato golden mosaic virus* (TGMV), mutants of AC4 show no effect on the virus’s symptom development and infectivity [116]. On the other hand, the AC4 protein in some bipartite Old World *Begomovirus* may function as a suppressor of PTGS by binding to miRNAs and impeding plant growth [117,118]. This shows that the function of AC4 protein is diverse and depends on the type of virus.

#### 3.1.5. Functional Studies on Betasatellite DNAβ of Monopartite Begomovirus

Monopartite *Begomovirus* genome mimics the DNA-A from bipartite *Begomovirus* [116]. In recent years, DNAβ, a symptom modulating betasatellites, has been associated with monopartite *Begomovirus* [119,120]. DNAβ is about 1.3–1.4 kb in size. Approximately half the virus genome’s size DNAβ carries a βC1 gene that is conserved, can suppress RNA silencing and hold together to ssDNA dsDNA in vitro in a sequence non-specific manner [121]. A study on the *Tomato leaf curl China virus* (ToLCCNV) by Yang et al. [122] found that this virus is related to a betasatellite molecule (ToLCCNB). TolCCNV is inoculated on *Nicotiana benthamiana* and no symptoms were observed. However, the plant begins to show a downward leaf curling symptom when transfected with ToLCCNV and ToLCCNB. This infectivity test showed that ToLCCNB is needed for the symptoms to appear. Also, the DNAβ with a mutated βC1 retains its capability for silencing suppressor, no typical symptom in *Nicotiana benthamiana* is observed when it is coinfected with ToLCCNV [122]. Table 2 shows the summary of functional studies conducted on *Begomovirus* genome.

### 3.2. Infectious Clones for Screening Resistant Varieties

One of the approaches to managing and controlling *Begomovirus*-causative diseases is based on the viral vector’s control, which is the whitefly (*Bemisia tabaci*) even this approach is known to be laborious [123]. Without infectious clones, researchers need to maintain a virus-free whitefly colony and propagate a certain whitefly biotype that transmits the plant’s disease and about 1500 whiteflies per plant are needed to successfully transmit *African cassava mosaic virus* (ACMV) clones into the cassava plant [124]. This indicates that a vast number of whiteflies are required to transmit the clone into the plant successfully. A previous study has reported that the whitefly transmission is controlled by selecting preferences of the vector and biotype compatibility with tested plants [125]. Additionally, wide-ranging screening genotypes of cassava for resistance to whitefly transmission has also shown favourable whitefly manifestation among cassava genotypes [126]. To deploy a resistant variety, early screening for resistance in the breeding line is desirable. The availability of infectious virus clones without depending on the natural infestation of viruliferous whiteflies could help early screening for the resistant varieties. Hence, resistant cultivar has become the best alternative for disease control [98,127].

As for early screening for resistant variety, biolistic inoculation was used to deliver cloned virus into cassava plant infection, speeding up evaluating cassava cultivars’ tolerance in the breeding program. The usage of biolistic has been reported to help determine the resistance level to cassava mosaic disease in cassava breeding lines [128]. The biolistic inoculation of DNA *Cassava mosaic virus* (*Begomovirus*) is more rapid in drawing out the infection symptom, which is as early as 10 days post-inoculation. Moreover, successfully infected plants produced are more significant in number. Hence, this indicates that using biolistic mediated transmission is more efficient than the whitefly-transmission technique [123,128].

## 4. Future Perspectives

For decades, the assembly of DNA constructs and viral clones has been conducted using the use of restriction endonucleases to create compatible ends. The presence or absence of restriction sites in the viral genome and vector sequences prevented this approach from being successful. Plant virologists have adopted several cloning methods to overcome these limitations such as overlap-based methods, Gibson assembly, Golden Gate cloning and yeast homologous recombination. Overlap-based methods are versatile, flexible and overcome the major constraints associated with the use of restriction enzymes. Overlap-based cloning allows for the rapid and efficient assembly of infectious clones. A simple approach to obtain infectious clones of the *Bean golden mosaic virus* was demonstrated using the PCR–Gibson Assembly technique [129]. The dimeric constructs that contain two replication origins are needed for each genomic component of *Begomovirus* infectious clones, and the single-step GA was successfully used to assemble the genome of the virus in an isothermal reaction. Golden Gate cloning and yeast homologous recombination has not yet been reported in the development of infectious clones in begomoviruses, and this might be due to their small genomes and cloning of the genome being relatively easy.

The potential of synthetic biology has been demonstrated through the development of functional and artificial genomes. In particular, the assembly of synthetic replicons from virus genomes has been shown to be an effective method for de novo synthesis. Several infectious clones from plant virus’s genomes have been reported to have been synthesized entirely using de novo synthesis and assembly approach [130,131,132]. In *Begomovirus*, the presence of conserved sequences allows the *Begomovirus* to utilize its degenerate “universal” primers to amplify the entire genome, even from total plant DNA. Genotypic sequences of begomoviruses were generated through a viral metagenomic approach using RCA–NGS (rolling circle amplification–next generation sequencing). The method was described to enable the analysis of the diversity of begomoviruses in North America, and 19 complete genome begomoviruses and one alphasatellite were assembled [133]. With the use of next-generation sequencing technology, the authenticity and reproducibility of begomoviral sequences will be verified by comparing them to those obtained using conventional methods such as PCR and RCA.

Cell-free cloning techniques have been used to obtain infectious clones from plant viruses [134,135]. Uncloned genome copies can be obtained by in vitro amplification and the products will be used to inoculate plants by rubbing or biolistic approach. This method is typically not suitable for reverse genomic studies [136]. The discovery that Agrobacterium can successfully infect plants with infectious clones opens new avenues of study in plant virology. As for delivering plants DNA or RNA viruses, agro-inoculation provides the most efficient and universal method. Its potential is greatly increased by the use of synthetic biology strategies [137,138]. Full-length infectious clones can be engineered to be optimized for industrial applications, sources of biomaterial and nanotechnology tools and viral vectors [132].

## 5. Conclusions

The availability of infectious clones for begomoviruses has proven to be a powerful molecular tool. This technique allows the functional study of a viral gene and its biological properties, and facilitates the genetic screening of germplasm for resistance to viruses. The agroinoculation of viral clones using *Agrobacterium tumefaciens* and its binary vectors is a cost-effective and highly efficient method for analyzing the *Begomovirus*’s infectivity. Thus, it is widely used by virologists. To date, the processes for producing a full-length infectious clone construct have been simplified and continuous developments of kits and methodologies have been made available to allow a better understanding of viral infectivity.

## Figures and Tables

**Table 1:** Compilation of strategy on cloning an infectious clone for *Begomovirus*.

Genome Structure	Virus	Genome Amplification	Cloning Vector	Transformation Method	Plant (Description)	Reference
Monopartite	*Ageratum enation virus* (AgEV)	RCA	pCambia2301	Agroinoculation	*Amaranthus cruentus* (severe leaf curl, enation and yellow vein symptom)	[41]
					*N. benthamiana* (severe leaf curl, enation and yellow vein symptom)	
					*N. glutinosa* (severe leaf curl, enation and yellow vein symptom)	
Monopartite	*Ageratum yellow vein China virus* (AYVCNV)	RCA	pBin19	Agroinoculation	*N. benthamiana* (severe downward leaf curl, leaf crinkle, stunting and vein swelling)	[42]
					*N. glutinosa* (leaf curling and distortion)	
					*P. hybrida* (leaf curling and distortion)	
					*L. esculentum* (leaf curling and distortion)	
					*Ageratum conyzoides* (yellow vein)	
Monopartite	*Ageratum yellow vein Taiwan virus* (AYVTV)	PCR	pBinPLUS	Agroinoculation	*N. benthamiana* (vein yellowing, leaf curling)	[30]
Monopartite	*Cotton leaf curl virus* (CLCuV)	PCR	pBin19	Biolistic	Cotton (vein swelling, vein darkening, leaf curling, and enations)	[43]
					*N. benthamiana* (mild stunting and yellowing)	
Monopartite	*Euphorbia leaf curl virus* (EuLCV)	PCR	pBinPLUS	Agroinoculation	*P. hybrida* (severe leaf curling and crinkling, vein swelling and stunting)	[44]
					*N. benthamiana* (severe upward leaf curling, vein swelling, and stunting)	
					*N. glutinosa* (vein swelling, stunting and downward leaf curling)	
					*Solanum lycopersicum* (severe leaf crinkling, vein swelling and stunting)	
Monopartite	*Grapevine red blotch virus* (GRBV)	RCA	pCambia2301	Agroinoculation	*Chenopodium album* (symptomless)	[45]
					*Lycopersicon esculentum* (symptomless)	
Monopartite	*Malvastrum leaf curl Guandong virus* (MLCuGdV)	PCR	pBinPLUS	Agroinoculation	*N. benthamiana* (yellow vein, leaf curling, vein swelling and stunting symptoms)	[46]
					*N. glutinosa* (leaf curling, vein swelling and stunting)	
					*P. hybrida* (leaf curling, vein swelling and stunting)	
Monopartite	*Malvastrum yellow vein virus* (MYVV)	PCR	pLH9000	Agroinoculation	*N. benthamiana* (downward curling of the leaves, yellow vein and leaf crinkling)	[47]
					*N. glutinosa* (downward curling of the leaves, yellow vein and leaf crinkling)	
					*P. hybrida* (downward curling of the leaves, yellow vein and leaf crinkling)	
					*M. coromandelianum* (yellow vein)	
Monopartite	*Malvastrum yellow vein Yunnan virus* (MYVYNV)	PCR	pBinPLUS	Agroinoculation	*N. benthamiana* (yellow vein, vein thickening and upward leaf curl symptoms)	[48]
Monopartite	*Papaya leaf curl China virus* (PaLCuCNV)	PCR	pBinPLUS	Agroinoculation	*S. lycopersicum* (leaf downward curling and crinkling)	[49]
					*N. benthamiana* (leaf upward curling and crinkling)	
					*N. tabacum* (leaf curling and crinkling)	
					*N. glutinosa* (leaf downward curling and crinkling)	
					*P. hybrida* (leaf upward curling and crinkling)	
Monopartite	*Passion fruit chlorotic mottle virus* (PCMoV)	RCA	pJETt 1.2	Agroinoculation	*N. benthamiana* (chlorotic spots, mottle and growth impairment)	[50]
					*Arabidopsis thaliana* (mild symptom)	
					*Passiflora edulis* (mild symptom)	
Monopartite	*Stachytarpheta leaf curl virus* (StaLCV)	PCR	pBinPLUS	Agroinoculation	*N. benthamiana* (upward leaf curling, swelling of the veins and stunting)	[51]
					*N. tabacum* (upward curling and vein swelling)	
					*Lycopersicon esculentum* (mild leaf distortion, puckering and leaf crinkling)	
					*P. hybrida* (severe upward leaf curling and vein swelling)	
Monopartite	*Sweet potato leaf curl virus* (SPLCV)	PCR	pGEM-T easy	Biolistic	Sweet potatoes (mild leaf curl)	[52]
Monopartite	*Sweet potato leaf curl virus*-Jiangsu (SPLCV-JS) &	RCA	pPZP100	Agroinoculation	Sweet potato: golden vein, mosaic and leaf curling symptoms	[53]
					*I. setosa*: mosaic, chlorosis and severe downward leaf curling	
					*N. benthamiana* (downward leaf curling)	
Monopartite	*Tobacco curly shoot virus* (TbCSV)	PCR	pBinPLUS	Agroinoculation	*N. benthamiana* (severe upward leaf curl, vein swelling and stunting)	[54]
					*N. glutinosa* (downward leaves curling)	
					*N. tabacum* (mild downward curling, puckering of leaves and vein thickening)	
Monopartite	*Tobacco leaf curl Yunnan virus* (TbLCYNV)	PCR	pBinPLUS	Agroinoculation	*N. benthamiana* (severe upward leaf curling and vein thickening)	[55]
					*P. hybrida* (severe upward leaf curling and stunting)	
					*N. glutinosa* (slight leaf curling and vein thickening)	
Monopartite	*Tomato leaf curl Al-Batinah virus* (ToLCABV)	RCA	pCambia1301	Agroinoculation	*N. benthamiana* (downward leaf curl, leaf crumpling, vein swelling, interveinal yellowing, a reduced leaf size for young leaves and stunting of plants)	[56]
					Tomato (interveinal chlorosis, leaf curling or crumpling)	
Monopartite	*Tomato leaf curl Sudan virus* (ToLCSDV)	RCA	pGreen0029	Agroinoculation	*N. benthamiana* (vein swelling and severe upward leaf curling)	[57]
					Tomato (mild downward curling, crumpling and interveinal yellowing)	
Monopartite	*Tomato leaf curl virus* (ToLCV)	RCA	Pg-Gez3	Agroinoculation	*N. benthamiana* (stunting, leaf curling, vein thickening, stem deformation	[58]
					Tomato (upward curling, reduced size of new leaflets and mild vein yellowing)	
Monopartite	*Tomato leaf curl virus* (Philippines) (ToLCV-Ph)	PCR	pBI121	Agroinoculation	Tomato (curling)	[59]
					*N. benthamiana* (wrinkling)	
Monopartite	*Tomato leaf curl virus* (TYLCV)	PCR	pBISzYI	Agroinoculation	*N. benthamiana* (upward leaf curl and plant stunting)	[60]
					*N. glutinosa* (stunting and mild leaf curling)	
					Tomato (mild leaf curl)	
Monopartite	*Tomato twisted leaf virus* (ToTLV)	RCA	pCAMBIA1300	Agroinoculation	Tomato (size reduction of young leaves, chlorosis and leaf deformation)	[61]
					*N. benthamiana* (mosaic symptoms)	
Monopartite	*Tomato yellow leaf curl Sardinia virus* (TYLCSV)	PCR	pBin19	Agroinoculation	Tomato (downward leaf curl and rugosity)	[62]
					*N. benthamiana* (downward curling, bubbling and interveinal yellowing)	
Monopartite	*Tomato yellow leaf curl virus* (TYLCV)	PCR	pGreen0.24	Agroinoculation	Tomato seedlings (downward leaf curling, reduced leaf size and marginal leaf chlorosis)	[63]
					Turnip seedling (outward leaf curling, swelling of veins, thick and brittle leaves)	
					Watermelon seedlings (severe stunting, severe leaf curling, leaf necrosis)	
Monopartite	*Tomato yellow leaf curl virus* (TYLCV)	PCR	pLH7000	Agroinoculation	Tomato (leaf curling and distortion, yellowing of leaves and reduction in plant growth)	[64]
					*N. benthamiana* (leaf curling and distortion, yellowing of leaves and reduction in plant growth)	
Monopartite	*Tomato yellow leaf curl virus* (TYLCV)	PCR	pGEM-5Zf+	Agroinoculation	Tomato (stunted and erect, leaves curl upwards, crumple and distinctive interveinal chlorosis)	[65]
Monopartite	*Tomato yellow leaf curl virus* (TYLCV)	PCR	pGreen	Agroinoculation	Tomato (yellow leaf curl and stunting)	[66]
Monopartite	*Tomato yellow leaf curl virus* (TYLCV)	PCR	pBBR1MCS-5	Agroinoculation	*Solanum nigrum* (yellowing, leaf distortion, dwarfing)	[67]
					tomato (yellowing and distortion)	
					Common bean (yellowing)	
Monopartite	*Tomato yellow leaf curl virus* (TYLCV)	PCR	pBinPLUS	Agroinoculation	*N. benthamiana* (downward leaf curling and vein yellowing)	[68]
					*S. lycopersicum* (downward leaf curling, yellowing and vein yellowing)	
					*N. tabacum* (downward leaf curling)	
					*N. glutinosa* (downward leaf curling)	
					*P. hybrida* (upward leaf curling)	
Monopartite	*Tomato yellow leaf curl virus* (TYLCV)	RCA	pCambia0390	Agroinoculation	Tomato (leaf curling, yellowing, and stunting)	[31]
Monopartite	*Vernonia yellow vein virus* (VeYVV)	RCA	pCambia2301	Agroinoculation	*Vernonia cinerea* (vein yellowing)	[69]
Bipartite	*Bean golden yellow mosaic virus* (BGYMV)	PCR	pCGN1547	Agroinoculation	Bean plants (moderate vein clearing and epinasty symptoms)	[70]
					*N. benthamiana* (mild epinasty and leaf crumpling)	
Bipartite	*Clerodendrum golden mosaic China virus* (ClGMCNV)	RCA	pBinPLUS	Agroinoculation	*N. benthamiana* (leaf crumpling and stunting)	[71]
					*N. glutinosa* (chlorotic spots and leaf distortion)	
					*N. tabacum* (leaf distortion and stunting)	
					*Petunia hybrida* (symptomless)	
					*Solanum lycopersicum* (no infection)	
Bipartite	*Corchorus mottle virus* (CoMoV)	RCA	pBluescript SK+	Biolistic	*Sida rhombifolia (*systemic vein chlorosis, mottling and leaf deformation)	[72]
					*N. benthamiana* and tomato (symptomless)	
Bipartite	*Cotton leaf curl Multan betasatellite* (CLCuMB)	RCA	pCambia1300	Agroinoculation	*N. benthamiana* (downward leaf curling, leaf puckering, chlorosis, reduction of the leaf lamina and shortening of internodes) *Rumex nepalensis* (no symptom)	[73]
Bipartite	*Croton yellow vein mosaic virus* (CYVMV)	PCR	pCambia2300	Agroinoculation	*Nicotiana tabacum* (mild puckering and vein clearing) *N. benthamiana* (leaf curling, leaf rolling, and vein thickening) *N. glutinosa* (mild puckering on leaves)	[74]
Bipartite	*Cucurbit leaf crumple virus* (CuLCrV)	PCR	pZeroBlunt	Agroinoculation	Common bean (stunted growth, leaf curl and chlorosis) *N. benthamiana* (stunted growth, leaf curl and chlorosis) Pumpkin (stunted growth, leaf curl, leaf yellowing and mottling) Zucchini (stunted growth, leaf curl, leaf yellowing and mottling)	[75]
Bipartite	*East African cassava mosaic Cameroon virus* (EACMCV)	PCR	pSKDf	Biolistic	*N.benthamiana* (mosaic, leaf distortion and yellowing)	[76]
Bipartite	*Indian cassava mosaic virus* (ICMV)	PCR	pCambia1300	Agroinoculation	*N. benthamiana* (plant stunting, downward leaf curling, yellow-green mosaic leaves) *J. curcas* (downward leaf curling, yellow-green mosaic, serration, leaf size reduction and plant stunting)	[77]
Bipartite	*Jatropha mosaic virus* (JaMV)	RCA	pBluescript KS (−)	Biolistic	*Jatropha multifida* (distortion, mosaic and necrosis) *N. tabacum* (chlorotic mottle and mild leaf curling) *Phaseolus vulgaris* (mosaic, leaf deformation and stunting)	[78]
Bipartite	*Legume yellow mosaic virus* (LYMV)	RCA	pBin19	Agroinoculation	*N. bentahamiana* (no infection) Soybean (mild chlorotic spots)	[79]
Bipartite	*Mungbean yellow mosaic India virus* (MYMIV)	RCA	pCambia3300	Agroinoculation	Cowpea (stunted growth, severe leaf curling, reduced leaf size, yellow patches and distortion of leaf lamina) Mungbean (stunted growth, severe leaf curling, reduced leaf size, yellow patches and distortion of leaf lamina)	[80]
Bipartite	*Mungbean yellow mosaic virus* (MYMV)	PCR	pBin19	Agroinoculation	Blackgram (mosaic pattern) Mungbean (mosaic pattern) French bean (backward leaf curling and stunting) Cowpea (severe leaf curl and leaf distortion)	[81]
Bipartite	*Pedilanthus leaf curl virus* (PeLCV)	RCA	pCambia2300	Agroinoculation	*N. benthamiana* (severe downward curling, swelling of leaves and vein yellowing) *Petunia atkinsiana* (upward curling and swelling of leaves)	[82]
Bipartite	*Pepper yellow leaf curl Indonesia virus* (PepYLCIV)	RCA	pGreen11	Agroinoculation	*N. benthamiana* (leaf curling, yellowing, mottling and stunting) Tomato (no symptom)	[83]
Bipartite	*Potato yellow mosaic virus* (PYMV)	PCR	pBluescript SK (+)	Agroinoculation	*Capsicum annuum* (mild yellow mosaic and distortion) *Datura stramonium* (yellow spots and leaf distortion) *N. benthamiana* (mild chlorosis and curling) *N. tabacum* (mild chlorosis and curling) *Petunia hybrida* (mild chlorosis and leaf curling) *Solanum tuberosum* (bright yellow mosaic and distortion)	[84]
Bipartite	*Sida golden mosaic virus* (SiGMoV)	PCR	pBluescript KS (−) pLitmus28	Biolistic	Common bean (mild mosaic and stunting) Cotton (asymptomatic) *N. bethamiana* (mosaic, leaf distortion and stunting *N. tabacum* (asymptomatic) Sida santaremensis (chlorotic mottle) Tomato (asymptomatic)	[85]
Bipartite	*Sida golden yellow vein virus* (SiGYVV)	RCA	pBluescript II SK (+)	Agroinoculation	*N. benthamiana* (chlorosis, leaf curling and severe stunting) *Malvastrum coromandelianum* (no symptom) *Sidastrum micranthum* (mild yellow mosaics)	[86]
Bipartite	*Squash leaf curl China virus* (SLCCNV)	PCR	pBI121	Agroinoculation	Pumpkin (stunting and leaf curling)	[87]
Bipartite	*Tomato golden vein virus* (TGVV)	RCA	pCambia0380	Agroinoculation	Tomato (vein yellowing) *N. benthamiana* (vein yellowing)	[88]
Bipartite	*Tomato leaf curl Palampur virus* (ToLCHnV)	PCR	pGreen 0029	Biolistic	*N. benthamiana* (upward leaf curling and vein swelling) Muskmelon (leaves necrosis near the vein)	[89]
Bipartite	*Tomato yellow leaf curl Thailand virus* (TYLCTHV)	PCR	pBinPLUS	Agroinoculation	*N. benthamiana* (leaf curling and vein thickening) N. glutinosa (downward leaf curling) Solanum lycopersicum (downward leaf curling and mild yellowing)	[90]
Bipartite	*Tomato yellow leaf distortion virus* (ToYLDV)	RCA	pBluescript SK pGEM-T easy	Biolistic	Tomato (yellowing and distortion) Soybean (no symptom) *N. tabacum* (yellowing and distortion) *N. benthamiana* (yellowing and distortion)	[91]
Bipartite	*Velvet bean severe mosaic virus* (VbSMV)	RCA	pCambia1300	Agroinoculation	*N. benthamiana* (downward leaf curling. Mottling and puckering) *N. glutinosa* (downward leaf curling, mottling and puckering)	[92]
Bipartite	*Watermelon chlorotic stunt virus* (WmCSV)	RCA	pCambia2300	Agroinoculation	*N. benthamiana* (mild disease symptoms) Watermelon (mottling, yellowing and severe leaf curling)	[93]
Bipartite	*Wissadula golden mosaic St Thomas virus* (WGMSTV)	PCR	pCR2.1-TOPO	Biolistic	Red pea (yellowing) *Wissadula amplissima* (blistering, yellow patches) *N. benthamiana* (blistering, yellowing) *Lycopersicon esculentum* (chlorosis)	[94]

**Table 2:** Summary of functional studies conducted on the genome of *Begomovirus*.

Gene Study	Virus	Strategy	Impact	Reference
AV2	*Tomato leaf curl Palampur virus* (ToLCPalV)	AV2 mutant infectious clones	Systematic infection occurs in *N. benthamiana and S. lycopersicum*	[103]
	*Okra enation leaf virus* (OELV)	AV2 mutant infectious clones	No necrotic and leaf curling symptom is observed in *N. benthamiana*	[104]
AC1	*African Cassava mosaic virus* (ACMV)	RXL mutant infectious clones	No symptom induction in *N. benthamiana.*	[105]
C2	*Bhendi yellow mosaic virus* (BYMV)	C2 mutant infectious clones	Abolished symptom and reduced viral load in *N. benthamiana.*	[107]
AC4	*African Cassava mosaic virus* (ACMV)	AC4 mutant infectious clones	Full symptom and infectivity on the infected plants.	[114,115]
	*Tomato golden mosaic virus* (TGMV)	AC4 mutant infectious clones	No infection and symptomless effect on the infected *N. benthamiana.*	[116]
BC1	*Tomato leaf curl China virus* (ToLCCNV)	ToLCCNB infectious clones	Asymptomatic infection occurs in *N. benthamiana.*	[122]

## Data Availability

No new data were created or analyzed in this study. Data sharing does not apply to this article.
